# Rac1 S71 Mediates the Interaction between Rac1 and 14-3-3 Proteins

**DOI:** 10.3390/cells8091006

**Published:** 2019-08-30

**Authors:** Abdalla Abdrabou, Daniel Brandwein, Changyu Liu, Zhixiang Wang

**Affiliations:** Department of Medical Genetics, and Signal Transduction Research Group, Faculty of Medicine and Dentistry, University of Alberta, Edmonton, AB T6G 2H7, Canada

**Keywords:** 14-3-3, isoforms, Rac1, interaction, phosphorylation, subcellular localization, Rac1 activity, binding

## Abstract

Both 14-3-3 proteins (14-3-3s) and Rho proteins regulate cytoskeleton remodeling and cell migration, which suggests a possible interaction between the signaling pathways regulated by these two groups of proteins. Indeed, more and more emerging evidence indicates the mutual regulation of these two signaling pathways. However, all of the data regarding the interaction between Rac1 signaling pathways and 14-3-3 signaling pathways are through either the upstream regulators or downstream substrates. It is not clear if Rac1 could interact with 14-3-3s directly. It is interesting to notice that the Rac1 sequence ^68^RPLSYP^73^ is likely a 14-3-3 protein binding motif following the phosphorylation of S71 by Akt. Thus, we hypothesize that Rac1 directly interacts with 14-3-3s. We tested this hypothesis in this research. By using mutagenesis, co-immunoprecipitation (co-IP), Rac1 activity assay, immunoblotting, and indirect immunofluorescence, we demonstrate that 14-3-3s interact with Rac1. This interaction is mediated by Rac1 S71 in both phosphorylation-dependent and -independent manners, but the phosphorylation-dependent interaction is much stronger. Epidermal growth factor (EGF) strongly stimulates the phosphorylation of Rac1 S71 and the interaction between 14-3-3s and Rac1. Mutating S71 to A completely abolishes both phosphorylation-dependent and -independent interactions between 14-3-3s and Rac1. The interaction between 14-3-3s and Rac1 mostly serve to regulate the activity and subcellular localization of Rac1. Among the seven 14-3-3 isoforms, 14-3-3η, -σ, and -θ showed interactions with Rac1 in both Cos-7 and HEK 293 cells. 14-3-3γ also binds to Rac1 in HEK 293 cells, but not in Cos-7 cells. We conclude that 14-3-3s interact with Rac1. This interaction is mediated by Rac1 S71 in both phosphorylation-dependent and -independent manners. The interaction between 14-3-3 and Rac1 mostly serves to regulate the activity and subcellular localization of Rac1. Among the seven 14-3-3 isoforms, 14-3-3η, -γ, -σ, and -θ interact with Rac1.

## 1. Introduction

Rac1 is a small GTPase (molecular mass 21,450 Da) that belongs to the Rho family of GTPases [1,2]. There are three members (Rac1, Rac2, and Rac3) in the Rac subfamily of the Rho family, and they share significant sequence similarity (~88%). These three Rac proteins diverge primarily in the C-terminal 15 residues. Similar to other Rho GTPases, the most studied function of Rac1 is the regulation of cytoskeleton remodeling through the regulation of actin and microtubules [2,3,4,5]. In particular, Rac1 stimulates the formation of lamellipodia and membrane ruffles. Rac1 promotes lamellipodia formation by stimulating the polymerization of branched actin at the leading edge of the cell [3]. Rac1 is also involved in the regulation of cell size, cell proliferation, cell survival, membrane trafficking, cell motility, polarity, and adhesion [2,5,6,7]. Similar to other small GTPases, Rac1 functions as a switch by cycling between inactive (GDP-bound) and active (GTP-bound) forms. The cycle between inactive and active forms is controlled by three classes of regulatory proteins: guanine nucleotide exchange factors (GEFs) that activate Rho GTPases by promoting the release of GDP to allow binding of GTP; GTPase-activating proteins (GAPs) that inactivate Rho GTPases by stimulating their intrinsic GTP-hydrolysis activity; and guanine-nucleotide-dissociation inhibitors (GDIs), which interact with the GDP-bound inactive Rho to prevent its exchange to the GTP-bound form and prevent its translocation to the membrane for action [7,8]. The cycling of Rac1 between the GTP- and GDP-bound states is essential for effective signal flow to elicit downstream biological functions [9,10]. Other modifications including RNA splicing [11,12], microRNAs [13], and various post-translational modifications have also been shown to regulate the activity and function of Rac1. The reported post-translational modifications include lipidation, ubiquitination, phosphorylation, and adenylylation [14]. All these post-translational modifications have been shown to play important roles in the regulation of Rac1 and other Rho GTPases [5,15]. Recent findings suggest that Rac1 is phosphorylated at multiple sites and the phosphorylation may play significant roles in regulating Rac1 functions [5,16]. Rac1 is phosphorylated on S71 by Akt [17]. This phosphorylation of Rac1 inhibits its GTP binding activity without any significant change in GTPase activity. Both the GTP-binding and GTPase activities of the mutant Rac1 S71A (Rac1 with the replacement of S71 by A) are abolished regardless of the activity of Akt [17]. It was later reported that the phosphorylation of Rac1 S71 decreases the pathogenic effect mediated by Clostridium difficile toxin A (TcdA) [18]. Moreover, phosphorylation of Rac1 at S71 represents a reversible mechanism to determine the binding specificity of Rac1/Cdc42 to their downstream substrates [19]. In addition, Rac1 is phosphorylated at Y64 by FAK and SRC kinases. Y64 phosphorylation targets Rac1 to focal adhesions. Rac1-Y64F displayed increased GTP-binding, increased association with βPIX, and reduced binding with RhoGDI as compared with wild-type Rac1 (Rac1-WT). Rac1-Y64D had less binding activity to PAK than Rac1-WT or Rac1-Y64F [20]. We have shown that ERK phosphorylates Rac1 T108 in response to EGF stimulation. This phosphorylation alters Rac1 activity, its interaction with PLC-γ1, and its subcellular localization and affects Rac1 function in mediating cell migration [21]. These findings demonstrate that both serine/threonine and tyrosine phosphorylation of Rac1 are common phenomena and regulate multiple aspects of Rac1 functions.

The family of 14-3-3 proteins (14-3-3s) comprise seven isoforms and exist as homo- and/or heterodimers in cells. 14-3-3s are 28–33 kDa, acidic, evolutionarily conserved, widely expressed proteins that bind to a vast number of intracellular proteins in normal and cancer cells [22,23]. 14-3-3s bind to two phosphorylation-dependent high affinity binding motifs: RSXpSXP (mode I) and RXY/FXpSXP (mode II) [24,25]. In addition to the two binding motifs, 14-3-3s exhibit binding to the extreme C-terminus (pSX_1–2_–COOH) of many proteins, lately defined as mode III [26,27,28]. By binding with serine/threonine phosphorylated intracellular proteins, they alter the conformation, activity, and subcellular localization of their binding partners [29,30,31,32]. 14-3-3s interact with a wide range of proteins involved in cell signaling, cytoskeleton remodeling, DNA repair, and transcription regulation. Therefore, 14-3-3s regulate diverse cell functions including cell cycle, cell development, cell proliferation, apoptosis, and cell motility. Recently, a growing number of proteins involved in actin remodeling have been identified as 14-3-3 binding partners [28,31,33,34]. Global down-regulation of 14-3-3 expression causes tumor suppression, while overexpression of 14-3-3s is often seen in many cancerous phenotypes [35,36,37,38,39,40].

Both 14-3-3s and Rho proteins regulate cytoskeleton remodeling and cell migration, which suggests a possible interaction between the signaling pathways regulated by these two groups of proteins. Indeed, more and more emerging evidence indicates the mutual regulation of these two signaling pathways [2]. Most findings support the role of 14-3-3s in the regulation of Rho GTPases by interacting with Rac1 regulators including GEFs, GAPs, and GDIs. Some early data also suggest that 14-3-3s could act downstream of Rac1 by modulating Rac1 substrates [2]. Thus far, all data regarding the interaction between Rac1 signaling pathways and 14-3-3 protein signaling pathways are through either the upstream regulators or downstream substrates. However, it is possible that Rac1 could interact with 14-3-3s directly. Here, we show that Rac1 interacts with 14-3-3s directly in response to EGF, and this interaction is mediated by the phosphorylation of Rac1 S71. This interaction was not dependent on Rac1 activity but slightly modulates Rac1 activity. Moreover, this interaction alters the subcellular localization of Rac1. Among the seven 14-3-3 isoforms, 14-3-3η, -σ, and -θ showed interactions with Rac1 in both Cos-7 and HEK 293T cells. 14-3-3γ also binds to Rac1 in HEK 293T cells, but not in Cos-7 cells.

## 2. Materials and Methods

### 2.1. Cell Culture and Treatment

COS-7 and 293T cells were cultured in DMEM (Dulbecco’s Modified Eagle’s Medium) supplemented with 10% fetal bovine serum (FBS), 100 IU/mL penicillin and 100 μg/mL streptomycin, and were maintained in a 5% CO_2_ atmosphere at 37 °C. For the EGF treatments, COS-7 cells were serum-starved for 12–16 h followed by addition of EGF to a final concentration of 50 ng/mL for 15 min or as indicated. For the treatment with BV02, cells were incubated with BV02 at the indicated concentration for 24 h. For wortmannin treatment, the cells were incubated with wortmannin at 100 nM for 30 min prior to EGF treatment.

### 2.2. Transient Transfection

COS-7 and 293T cells were grown to 70–80% confluency in 6-cm dishes before the transfection. The transfection was performed using the calcium phosphate transfection method with BES buffer (40 mM NaCl, 0.75 mM sodium phosphate dibasic (Na_2_HPO_4_), 25 mM BES, pH 6.95). Cells were typically analyzed 36–48 h post-transfection.

### 2.3. Antibodies and Chemicals

Mouse monoclonal anti-Rac1 antibody was purchased from Cytoskeleton Inc. (Denver, CO, USA). Rabbit anti-GFP antibody was from Clontech (Mountain View, CA, USA). Mouse monoclonal anti-pRac1S71, anti-14-3-3s, 14-3-3η, -γ, -σ, and -θ, and anti-GST antibodies were from Santa Cruz Biotechnology, Inc. (Santa Cruz, CA, USA). Rabbit monoclonal anti-α-tubulin antibody was from Abcam (Abcam Inc, Toronto, ON, Canada). FITC- and TRITC-conjugated donkey anti-mouse and anti-rabbit antibodies were from Jackson ImmunoResearch Laboratories, Inc. (West Grove, PA, USA). Glutathione cross-linked to 4% agarose, goat anti-mouse IgG conjugated with agarose, and protein A conjugated with agarose were purchased from Sigma-Aldrich (St. Louis, MO, USA). Mammalian Protein Extraction Reagent (M-Per) was purchased from Thermo Fisher Scientific Inc. (Rockford, IL USA). Unless otherwise specified, all chemicals were purchased from Sigma-Aldrich.

### 2.4. Plasmids

A plasmid encoding GFP-Rac1 was a gift from Dr. Mark R. Philips (New York University, New York, NY, USA). A plasmid encoding GST-PAK was a gift from Dr. Gary Eitzen (University of Alberta, Edmonton, AB, Canada). Plasmids encoding GST-Rac1, GFP-tagged constitutively active Rac1 L61 (GFP-L61) and dominant-negative Rac1 N17 (GFP-N17) were generated previously in the laboratory [21,41]. GFP- and GST-tagged mutant Rac1 S71A were created with the QuikChange Multiple Site-directed Mutagenesis Kit (Stratagene, La Jolla, CA, USA) using GFP-Rac1 as a template.

### 2.5. Expression and Purification of GST-Fusion Proteins

To purify various GST-fusion proteins, the pGEX plasmids containing GST alone, GST-Rac1, GST-PAK, and GST-S71 constructs were transformed into *Escherichia coli* DH5α. Bacteria were grown to an optical density (OD)_600_ of 0.6–0.8 at 37 °C and induced with 0.2 mM isopropyl-1-thio-β-d-galactopyranoside (IPTG) and incubated for 4 h at 30 °C with shaking. After pelleting, bacterial cells were lysed by sonication in PBS in the presence of protease inhibitors (0.1 mM 4-(2-aminoethyl)-benzenesulfonyl fluoride, 10 μg/mL aprotinin, and 1 μM pepstatin A). After sonication, 1% Triton X-100 was added to enhance solubilization. Particulates were removed by centrifugation for 15 min at 10,000 rpm and the cleared supernatant was incubated with 50:50 glutathione-agarose beads (Sigma-Aldrich) in PBS for 2 h at 4 °C. The beads were washed three times with ice-cold PBS and stored. The immobilized GST fusion proteins on the beads were used for GST pull-down assays.

### 2.6. GST Pull-Down Assay

COS-7 cells were lysed into BOS buffer (50 mM Tris-HCl, pH 7.4, 200 mM NaCl, 1% Nonidet P-40, 10% glycerol, 10 mM NaF, 2.5 mM MgCl_2_, and 1 mM EDTA) with protease inhibitors. The lysates were centrifuged at 21,000× *g* at 4 °C for 15 min. Supernatants were used in the pull-down assay. GST-fusion proteins bound to glutathione-agarose beads were added to the supernatant and incubated at 4 °C for 2 h with shaking. Beads were collected by centrifugation and washed three times with BOS buffer after which the 2× sample loading buffer was added. The pull-down proteins were resolved on SDS-PAGE and analyzed by Western blotting.

### 2.7. Rac1 Activity Assay

Rac1 activity was determined using an assay as we described previously [21,41]. The Rac1 binding domain of PAK, a Rac1 effector, was used as a GST fusion protein to pull down active Rac1. Briefly, COS-7 cells, with transfections, were lysed into GST-PAK buffer (50 mM Tris-HCl, pH 7.6, 150 mM NaCl, 1% Triton X-100, and 10 mM MgCl_2_) with protease inhibitors. The lysates were centrifuged at 21,000× *g* at 4 °C for 15 min. Supernatants were used in the binding assay. GST-PAK fusion proteins bound to glutathione-agarose beads in GST-PAK buffer were added and incubated at 4 °C for 2 h. Beads were collected by centrifugation, washed three times with GST-PAK buffer, after which SDS loading buffer was added. The pull-down active Rac1 were resolved on SDS-PAGE and analyzed by Western blotting.

### 2.8. Immunoprecipitation

IP experiments were carried out as described previously [34]. Briefly, cells were lysed with IP buffer (20 mM Tris, pH 7.5, 150 mM NaCl, 1% Nonidet P-40, 0.1% sodium deoxycholate, 100 mm NaF, 5 mM MgCl_2_, 0.5 mM Na_3_VO_4_, 0.02% NaN_3_, 0.1 mM 4-(2-aminoethyl)-benzenesulfonyl fluoride, 10 μg/mL aprotinin, and 1 μM pepstatin A). Cell lysates were centrifuged at 22,000× *g* for 30 min to remove debris. The supernatants, containing approximately 1 mg of total protein, were pre-cleared with the agarose beads and then were used to incubate with 1 μg of specific antibody at 4 °C overnight with gentle mixing. Then, goat anti-mouse IgG conjugated with agarose or protein A conjugated with agarose was added to each fraction and incubated for 2 h at 4 °C with agitation. Both the agarose beads and the non-precipitated supernatant were collected by centrifugation. For the controls, mouse or rabbit IgG was used to replace the primary antibodies. The agarose beads were washed three times with IP buffer and then mixed with 2× sample loading buffer. The sample was boiled for 5 min and subjected to the Western blot assay.

### 2.9. Immunoblotting

The protein content of cell lysates was determined by Bradford analysis, and approximately 20 μg of total protein was used for each sample. Protein samples were resolved by SDS-PAGE and electrophoretically transferred onto nitrocellulose membranes. After blocking in 3% milk for 60 min, membranes were incubated with primary antibody at 4 °C overnight. The primary antibodies were detected with their corresponding horseradish peroxidase-conjugated secondary antibodies followed by enhanced chemiluminescence development (Pierce Chemical, Rockford, IL, USA) and light detection with Fuji (Tokyo, Japan) Super RX film.

### 2.10. Fluorescence Microscopy

Cells were cultured on glass coverslips for 48 h before treatment. After treatment, the cells were rinsed in tris-buffered saline (TBS; 6% tris, 8.8% NaCl, 85.2% dH_2_O, pH 7.6) and were fixed by cold methanol for 4 min. Cells were permeabilized with TBS containing 0.2% Triton X-100 for 10 min followed by blocking with TBS containing 1% BSA and 0.1% Triton X-100 for an hour. After blocking, the coverslips were incubated in 1 µg/mL primary antibody in TBS with 0.1% Triton X-100 as indicated for an hour. Afterwards, the coverslips were rinsed in TBS with 0.1% Triton X-100 three times each for 5 min and then incubated in 1 µg/mL solution of FITC- and/or TRITC-conjugated secondary antibody in TBS with 0.1% Triton X-100 with for an hour in the dark. Thereafter, the coverslips were washed completely in TBS and incubated in 1 µg/mL of 4′,6-diamidino-2-phenylindole (DAPI) solution in TBS for 5 min at room temperature in the dark. The coverslips were then mounted on glass slides and observed using a DeltaVision fluorescence microscopy system (Applied Precision Inc., Mississauga, ON, Canada).

### 2.11. Artificial Network Analysis

Using the predictions generated from the Barton group 14-3-3 Pred, data were integrated from support vector machines (SVM), position-specific scoring matrices, and support vector machines (SVM) and artificial neural network (ANN) classification methods were trained to discriminate experimentally determined 14-3-3-binding motifs from non-binding phosphopeptides. Afterwards, we used Netphos to predict the molecule that is likely to be involved in the phosphorylation of S71. Then, we used R-studio to generate a blot graph representing the sites that are most likely in genuine interaction with Rac1. Rac1 S71 phosphorylation by PKB (AKT) showed the highest consensus among all other motifs and phosphorylation kinases.

### 2.12. Quantification and Statistical analysis

All protein bands were quantitated by densitometry using ImageJ software (ImageJ2, NIH, Bethesda, MD, USA). Data were statistically analyzed by one-way analysis of variance (ANOVA) using Prism V.8 software (GraphPad Software, La Jolla, CA, USA). Data are presented as mean and standard deviation. *p* < 0.05 “APA” was considered as statistically significant.

## 3. Results

### 3.1. Association between Rac1 and 14-3-3s in Response to EGF-Induced Akt Phosphorylation

**Figure 1:**
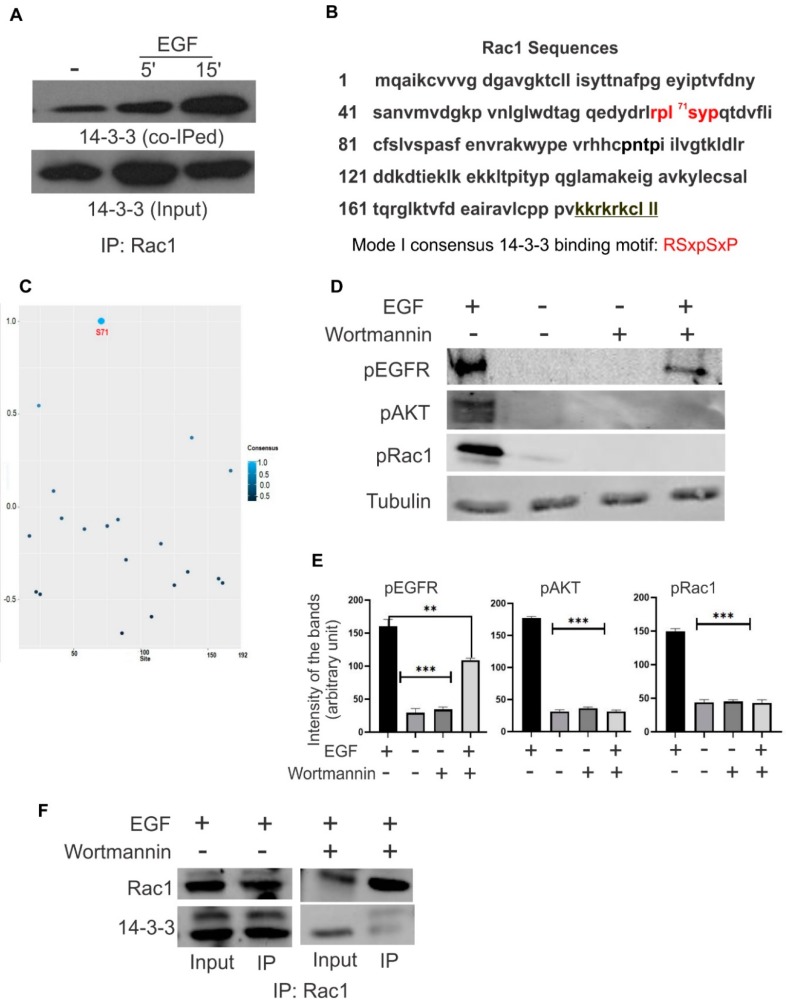
14-3-3s interact with Rac1 in response to EGF in Cos-7 cells. (**A**) 14-3-3s interact with Rac1 in response to EGF in Cos-7 cells. Cos-7 cells were treated with EGF for the indicated time and the interaction between 14-3-3s and Rac1 was determined by co-immunoprecipitation (co-IP) of 14-3-3s upon immunoprecipitation (IP) of Rac1. (**B**) Sequence analysis of Rac1 reveals the presence of the mode I 14-3-3 binding motif ^68^RPLSYP^73^ if S71 is phosphorylated. (**C**) The highest consensus between 14-3-3 and Rac1 is within the S71-containing motif as predicted by ANN 14-3-3 Prediction software. (**D**) The effects of EGF and wortmannin on the phosphorylation of EGFR, Akt, and Rac1 S71. Cos-7 cells were treated with EGF and/or wortmannin. The phosphorylation of EGFR, Akt, and Rac S71 was revealed by immunoblotting with phospho-specific antibodies. (**E**) Quantification of the data in (**D**). The level of protein phosphorylation were quantitated by densitometry. Each value is the average of at least three experiments and the error bar is standard error. ** *p* < 0.01, *** *p* < 0.001. (**F**) The effects of EGF and wortmannin on the interaction between 14-3-3s and Rac1. Cos-7 cells were treated with EGF and/or wortmannin as indicated. The interaction between 14-3-3s and Rac1 was determined by co-IP of 14-3-3s upon IP of Rac1.

More and more emerging evidence indicates the mutual regulation of Rac1 signaling pathways and 14-3-3 signaling pathways in terms of the regulation of cytoskeleton remodeling and cell migration in response to growth factor stimulation [2]. Here, we first determined whether Rac1 and 14-3-3s physically associate in response to EGF by co-immunoprecipitation (co-IP). Cos-7 cells were treated with EGF for the indicated time, and Rac1 was immunoprecipitated with mouse anti-Rac1 antibody. The co-IP of 14-3-3s was examined by immunoblotting with antibodies to pan 14-3-3 protein. As shown in Figure 1A, 14-3-3s co-immunoprecipitated (co-IPed) with Rac1 following EGF stimulation and the amount of co-immunoprecipitated 14-3-3s reached a maximum at 5–15 min following EGF stimulation. This indicates that EGF stimulates the association between Rac1 and 14-3-3s.

To understand the mechanisms underlying this interaction, we analyzed the amino acid sequence of Rac1 to identify potential binding motifs for 14-3-3 protein. The mode I consensus 14-3-3 protein binding motif is RSXpSXP, and the Rac1 sequence ^68^RPLSYP^73^ is likely a 14-3-3 protein binding motif following the phosphorylation of S71 (Figure 1B). It is well established that Rac1 is phosphorylated at S71 by Akt [17]. Moreover, ANN 14-3-3 Prediction software has predicted the highest consensus between 14-3-3 and Rac1 to be within the S71-containing motif, where S71 has a value of approximately 1 (Figure 1C). Thus, we propose that in response to EGF, Akt is phosphorylated by activated EGFR via PI3K, which results in the phosphorylation of Rac1 S71 and the interaction between Rac1 and 14-3-3s.

To test this hypothesis, we treated the cells with EGF and showed that EGFR, Akt and Rac1 S71 are all phosphorylated in response to EGFR (Figure 1D). However, inhibition of PI3K with wortmannin blocked the phosphorylation of both Akt and Rac1 S71 (Figure 1D). We then examined if wortmannin is able to inhibit the interaction between Rac1 and 14-3-3s. As shown in Figure 1E, wortmannin treatment inhibited the EGF-induced interaction between Rac1 and 14-3-3s. These data strongly suggest that EGF-induced Rac1 S71 phosphorylation through Akt leads to the interaction between Rac1 and 14-3-3s.

### 3.2. Rac1 and 14-3-3s Interaction is Mediated by Rac1 S71

**Figure 2:**
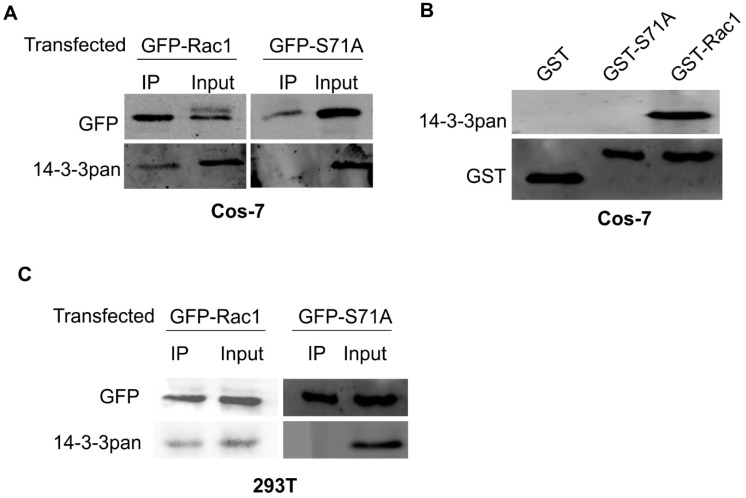
The effects of Rac1 S71 mutation to A on the interaction between 14-3-3s and Rac1 in both Cos-7 and 293T cells. (**A**) The effects of Rac1 S71 mutation to A on the interaction between 14-3-3s and Rac1 in Cos-7 cells. Cos-7 cells were transfected with wild type GFP-Rac1 and mutant GFP-S71A. The expressed Rac1 was immunoprecipitated (IPed) with an antibody to GFP, and the co-IPed 14-3-3s were examined by immunoblotting. (**B**) The effects of Rac1 S71 mutation to A on the interaction between 14-3-3s and Rac1 in Cos-7 cells by GST pulldown. The Cos-7 lysates were incubated with glutathione-agarose beads charged with GST, GST-Rac1 or mutant GST-S71A. The agarose beads were then separated and subjected to immunoblotting analysis with an antibody against 14-3-3 and GST. (**C**) The effects of Rac1 S71 mutation to A on the interaction between 14-3-3s and Rac1 in 293T cells. 293T cells were transfected with either GFP-Rac1 or GFP-S71A. GFP-Rac1 and GFP-S71A were IPed with the anti-GFP antibody. The co-IPed 14-3-3s were examined by immunoblotting.

To confirm the role of Rac1 S71 in mediating the interaction between Rac1 and 14-3-3s, we generated a GFP-tagged mutant Rac1 with the mutation of S71 to A (GFP-S71A). Our previously generated GFP-tagged wild type Rac1 (GFP-Rac1) was used as a control. We transfected Cos-7 cells with both GFP-Rac1 and GFP-S71A. As shown in Figure 2A, wild type Rac1 co-IPed with 14-3-3s. Intriguingly, the S71A mutant failed to co-IP with 14-3-3s, which supports the prediction that S71 phosphorylation is critical in mediating the interaction between Rac1 and 14-3-3s as previously described. To further validate our results, we also transfected 293T cells with GFP-Rac1 and GFP-S71A and we showed that GFP-Rac1, but not GFP-S71A, co-IPed with 14-3-3s (Figure 2C), which confirmed our observations in Cos-7 cells.

To further examine the interaction between Rac1 and 14-3-3s, we performed a GST pull-down experiment in Cos-7 cells. We generated GST-tagged Rac1 with the mutation of S71 to A (GST-S71A). We have generated GST-tagged wild type Rac1 (GST-Rac1) previously [41]. Lysates of COS-7 cells were incubated with glutathione-agarose beads charged with GST, GST-S71A or GST-Rac1. Following the incubation, the proteins that associated with the agarose beads were immunoblotted with antibodies to pan 14-3-3s. As shown in Figure 2B, while the detectable amount of 14-3-3s was pulled down by GST-Rac1, no 14-3-3s were pulled down by GST-S71A. This result indicates that the mutation of S71 to A completely inhibits the binding between Rac1 and 14-3-3s.

### 3.3. The Relationship between Rac1 Activity and Its Interaction with 14-3-3s

**Figure 3:**
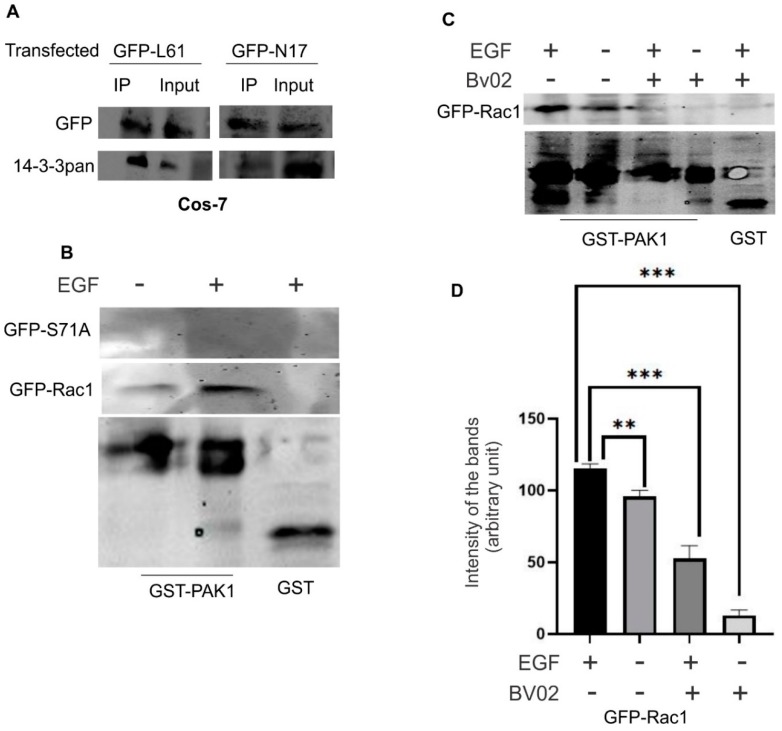
The relationship between Rac1 activity and its interaction with 14-3-3s in Cos-7 cells. (**A**) The effects of Rac1 activity on the interaction between 14-3-3s and Rac1. Cos-7 cells were transfected with either constitutively activated Rac1 (GFP-L61) or dominant-negative Rac1 (GFP-N17). The interaction of these two mutants with 14-3-3s was examined by co-IP as described above. (**B**) The effects of Rac1 S71 mutation to A on the activation of Rac1. Cos-7 cells were transfected with either GFP-Rac1 or mutant GFP-S71A, incubated with or without EGF for 15 min, and lysed. The cell lysates were incubated with GST fusion Rac-binding domain of PAK (GST-PAK) or GST bound to glutathione-agarose beads. The active Rac1 that binds to GST-PAK was determined by immunoblotting with an antibody to GFP; GST-PAK and GST were detected with an antibody to GST. (**C**) The effects of BV02 on the activation of Rac1. Cos-7 cells were transfected with GFP-Rac1 and incubated with BV02 at a concentration of 30 µM for 24 h. The cells were then incubated with or without EGF for 15 min. Cells were then lysed and the cell lysates were incubated with GST-PAK or GST bound to glutathione-agarose beads. The active Rac1 that binds to GST-PAK was determined by immunoblotting with an antibody to GFP; GST-PAK and GST were detected with an antibody to GST. (**D**) Quantification of the data in (**C**). The pulled-down active Rac1 bands were quantitated by densitometry. Each value is the average of at least three experiments and the error bar is standard error. ** *p* < 0.01, *** *p* < 0.001.

To examine if Rac1 activity has any effects on its interaction with 14-3-3s, we transfected Cos-7 cells with two commonly known GFP-tagged mutants; the constitutively active Rac1 (GFP-L61, with the replacement of 61Q by L) and the dominant-negative Rac1 (GFP-N17, with the replacement of 17T by N) that were generated previously [41]. The interaction between transfected Rac1 mutants and 14-3-3s was first examined by co-IP experiments. As shown in Figure 3A, both GFP-L61 and GFP-N17 co-IPed with 14-3-3s, similar to wild type GFP-Rac1 (Figure 1A), which indicates that Rac1 activity does not affect its interaction with 14-3-3s. We then examined if the interaction between Rac1 and 14-3-3 affects the activity of Rac1. As the mutation of S71 to A disrupts the interaction between Rac1 and 14-3-3, we examined the activity of GFP-S71A. We transfected Cos-7 cells with GFP-Rac1 and GFP-S71A, stimulating cells with EGF for 15 min, subjected the cell lysate to Rac1 activity assay using GST fusion Rac-binding domain of PAK (GST-PAK) or GST bound to glutathione-agarose beads. As shown in Figure 3B, disrupting the interaction between Rac1 and 14-3-3s by mutating S71 to A significantly reduced Rac1 activity. We then examined if disruption of the interaction between Rac1 and 14-3-3s, by inhibiting 14-3-3 docking sites, also affects Rac1 activity. BV02 is an inhibitor of the 14-3-3 scaffolding protein docking sites [42]. We treated Cos-7 cells with BV02 and examined the effects on Rac1 activity with or without EGF stimulation. As shown in Figure 3C,D, EGF stimulated the activation of Rac1 and BV02 inhibited Rac1 activity both with and without EGFR stimulation. Together, these data suggest that Rac1 activity has no effects on its interaction with 14-3-3; however, the interaction between Rac1 and 14-3-3 protein is important for EGF-induced Rac1 activation.

### 3.4. The Interaction between Rac1 and Various 14-3-3 Isoforms

**Figure 4:**
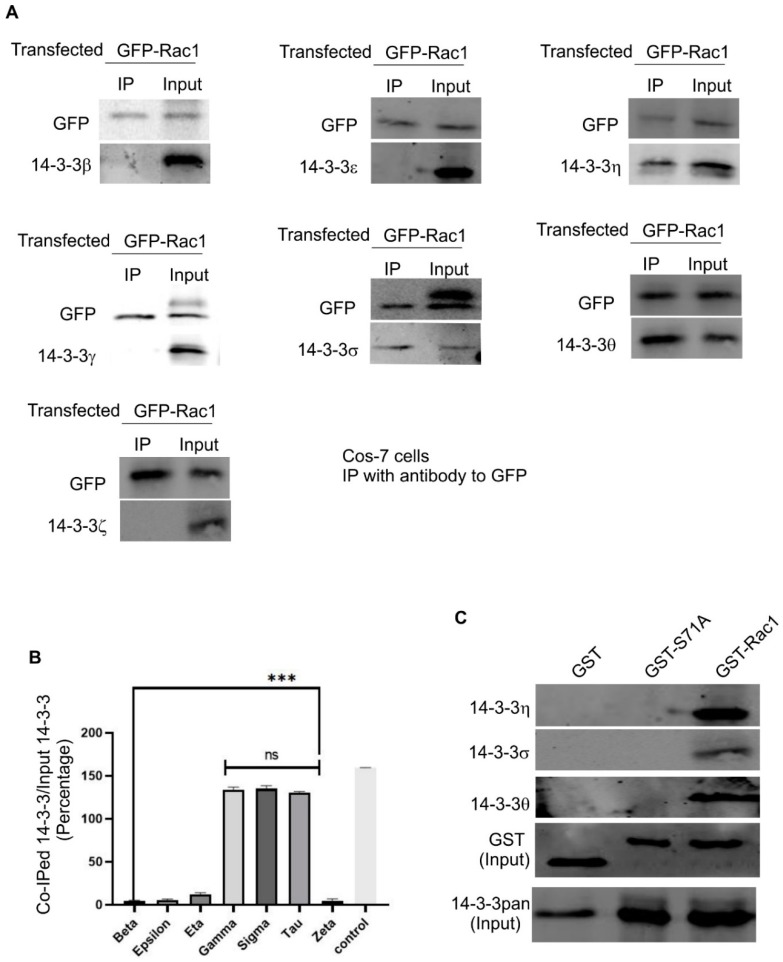
The interaction between Rac1 and seven 14-3-3 isoforms in Cos-7 cells. (**A**) Co-IP experiments to determine the interaction between Rac1 and seven 14-3-3 isoforms in Cos-7 cells. Cos-7 cells were transfected with GFP-Rac1. The expressed GFP-Rac1 was IPed with the anti-GFP antibody. The co-IP of various 14-3-3 isoforms was determined by immunoblotting. (**B**) Quantification of the data in (**A**). The levels of co-IPed 14-3-3s were quantitated by densitometry. Each value is the average of at least three experiments and the error bar is standard error. *** *p* < 0.001, ns: *p* > 0.1. (**C**) GST pull-down experiments to determine the interaction between three 14-3-3 isoforms including 14-3-3η, -σ, and -θ and GST-Rac1 and GST-S71A. The Cos-7 lysates were incubated with glutathione agarose beads charged with GST, GST-Rac1 or mutant GST-S71A. The agarose beads were then separated and subjected to immunoblotting analysis with antibodies against 14-3-3 η, -σ, and -θ, GST, and 14-3-3s.

There are seven 14-3-3 isoforms in mammalian cells. These isoforms have distinct subcellular localizations and functions [43]. We next examined which 14-3-3 isoform interacts with Rac1. We transfected Cos-7 cells with GFP-Rac1. Following the immunoprecipitation (IP) with an antibody to GFP, we examined which 14-3-3 isoforms co-IPed with GFP-Rac1 by immunoblotting with antibodies to various 14-3-3 isoforms. As shown in Figure 4A, 14-3-3η, -σ, and -θ co-IPed with GFP-Rac1, but none of 14-3-3β, -ε, -γ, and -ζ co-IPed with GFP-Rac1. To confirm these interactions and to determine if Rac1 S71 is responsible for these interactions, we performed GST-pulldown experiments with GST-Rac1 and GST-S71A. As shown in Figure 4B, GST-Rac1 was able to pull down all three identified isoforms including 14-3-3η, -σ, and -θ. However, GST-S71A was unable to pull down these isoforms. These data indicate the essential role of S71 in the interaction.

**Figure 5:**
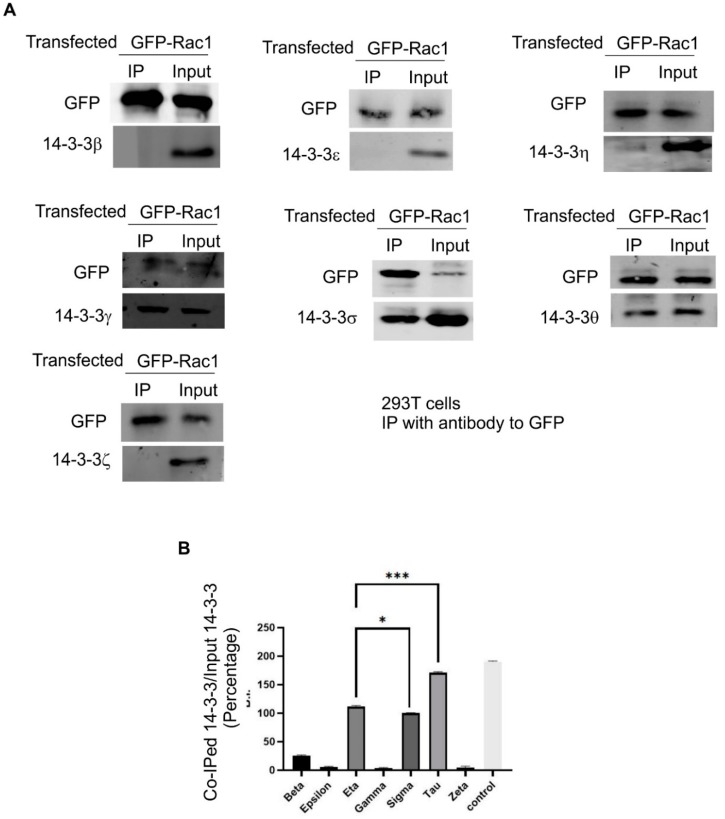
The interaction between Rac1 and seven 14-3-3 isoforms in 293T cells. (**A**) 293T cells were transfected with GFP-Rac1. The expressed GFP-Rac1 was IPed with the anti-GFP antibody. The co-IP of various 14-3-3 isoforms was determined by immunoblotting. (**B**) Quantification of the data in (**A**). The levels of co-IPed 14-3-3s were quantitated by densitometry. Each value is the average of at least three experiments and the error bar is standard error. * *p* < 0.1, *** *p* < 0.001.

We next examined the interaction between Rac1 and various 14-3-3 isoforms in 293T cells. We transfected 293T cells with GFP-Rac1. Following the IP with an antibody to GFP, we examined which 14-3-3 isoforms co-IPed with GFP-Rac1 by immunoblotting with antibodies to various 14-3-3 isoforms. As shown in Figure 5, we confirmed that, in 293T cells, 14-3-3η, -σ, and -θ also co-IPed with GFP-Rac1 as in Cos-7 cells; however, we observed that, differently from Cos-7 cells, 14-3-3γ co-IPed with GFP-Rac1 in 293T cells. As in Cos-7 cells, none of 14-3-3β, -ε, and -ζ co-IPed with GFP-Rac1. These data further indicate that Rac1 selectively interacts with a subset of 14-3-3 isoforms.

### 3.5. The Effects of Rac1 and 14-3-3 Protein Interaction on the Subcellular Localization of Rac1 and 14-3-3s

**Figure 6:**
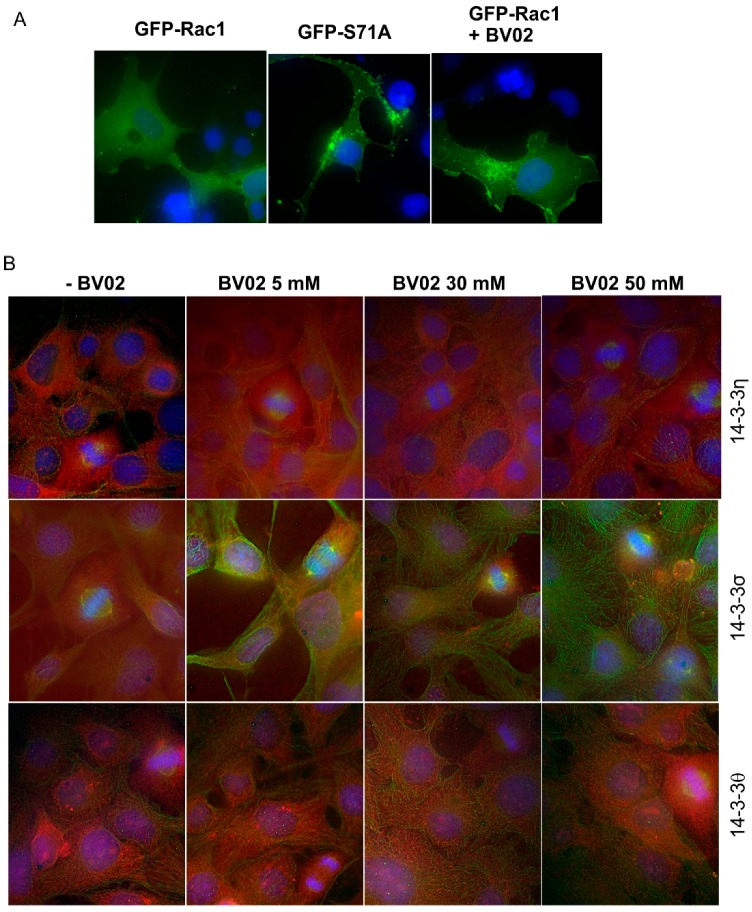
Disruption of the interaction between 14-3-3s and Rac1 and the effects on the subcellular localization of Rac1 and three 14-3-3 isoforms including 14-3-3η, -σ, and -θ. (**A**) The effects on the subcellular localization of Rac1. COS-7 cells were transfected with GFP-Rac1 or GFP-S71A. With or without treatment with BV02 for 24 h, the subcellular localization of GFP-Rac1 and GFP-S71A was revealed by the intrinsic fluorescence (green). The cells were counterstained with DAPI (blue). Size bar = 10 µm. (**B**) The effects on the subcellular localization of 14-3-3 isoforms. Cos-7 cells were treated with BV02 at the indicated concentration for 24 h. The cells were stained with mouse antibodies to 14-3-3η, -σ, and -θ and a rabbit antibody to α-tubulin, followed by incubation with TRITC-conjugated anti-mouse IgG and FITC-conjugated anti-rabbit IgG. DNA was stained by DAPI. The localization of 14-3-3 isoforms is shown as red, α-tubulin as green and chromosome/nucleus as blue. Size bar = 10 µm.

We finally examined if the interaction between Rac1 and 14-3-3s has any effect on the subcellular localization of both proteins. We first examined the effects on the subcellular localization of Rac1 following the disruption of the interaction between Rac1 and 14-3-3s. We transfected the Cos-7 cells with GFP-Rac1 and GFP-S71A and the localization of these two proteins were revealed by their intrinsic fluorescence. As shown in Figure 6A, GFP-Rac1 is mostly diffusively localized to the cytoplasm, with visible plasma membrane localization; however, GFP-S71A was mostly localized to the plasma membrane and associated with punctate structures with a much weaker cytosolic distribution. We also transfected the Cos-7 cells with GFP-Rac1 and then treated the cells with BV02 to disrupt the interaction between Rac1 and 14-3-3s. As shown in Figure 6A, in the presence of BV02, GFP-Rac1 showed similar subcellular localization as the mutant GFP-S71A as it mostly associated with the plasma membrane and showed punctate structures with a much weaker cytosolic distribution. These data strongly indicate that the interaction between Rac1 and 14-3-3 protein plays an important role in regulating the subcellular localization of Rac1.

We also examined the effects of BV02 on the subcellular localization of the three 14-3-3 isoforms. As shown in Figure 6B, there are no significant changes in the subcellular localization of these 14-3-3 isoforms following the treatment of the cells with BV02 at various concentrations. Thus, disrupting the interaction between 14-3-3 and Rac1 did not alter the subcellular localization of 14-3-3s.

## 4. Discussion

It is well-established that 14-3-3 protein signaling pathways and Rac1 signaling pathways co-regulate important cell functions including cytoskeleton remodeling and cell migration. The interaction is mostly through the interactions between 14-3-3s and Rac1 upstream regulators or downstream substrates [2]. Here, we demonstrated that a subset of 14-3-3s are able to interact directly with Rac1, which provides an additional regulation of Rac1 signaling pathways by 14-3-3 protein.

Both sequence analysis and ANN 14-3-3 Prediction software suggest that Rac1 S71 constitutes a 14-3-3 binding motif following its phosphorylation. In fact, the Rac1 sequence ^68^RPLSYP^73^ is likely the mode I 14-3-3 consensus binding motif following the phosphorylation of S71 (Figure 1). It is well established that Rac1 is phosphorylated at S71 by Akt [17]. We showed in this research that EGF stimulated the phosphorylation of Akt and Rac1 S71, which leads to the interaction between 14-3-3 and Rac1. Inhibition of Akt phosphorylation by wortmannin inhibited the EGF-induced interaction between 14-3-3s and Rac1 (Figure 1). Moreover, we showed that the mutation of S71 to A inhibited the interaction between 14-3-3s and Rac1. These data strongly indicate the importance of S71 and its phosphorylation in mediating the interaction between 14-3-3s and Rac1 (Figure 2).

While it has been shown that Akt phosphorylates Rac1 [17], little is known about the functional significance of this interaction. Our findings here indicate that the phosphorylation of S71 creates a strong binding motif for 14-3-3s, which allows for the direct interaction between 14-3-3s and Rac1. As both 14-3-3s and Rac1 regulate many common cell functions, our findings suggest the presence of another layer of regulation.

Interestingly, while our co-IP experiments indicate that the interaction between 14-3-3s and Rac1 is dependent on EGF stimulation and subsequent Rac1 S71 phosphorylation (Figure 1 and Figure 2), the GST pull-down experiments suggest that 14-3-3s may interact with Rac1 in the absence of S71 phosphorylation as GST-Rac1 pulled down 14-3-3s (Figure 2B and Figure 4B). The inability to pull down 14-3-3s by GST-S71A indicates the requirement of S71 for the interaction between 14-3-3s and Rac1. Although most interactions between 14-3-3s and their binding partners are mediated by phosphorylated motifs of the binding partners, a significant number of proteins have been shown to interact with 14-3-3s in a phosphorylation-independent manner. These proteins include human telomerase (hTERT) [44], SGK1 and tau [45], amyloid β-protein precursor intracellular domain fragment [46], HSP60 and PrP^C^ [47], the enteropathogenic *Escherichia coli* Tir protein [48], exoenzyme S [49], NtCDPK1 [50], non-muscle myosin II [51], liver kinase B1(LKB1) [52,53], and RGS14 [54]. Among these proteins, SGK1, LKB1, NtCDPK1, and RGS14 interact with 14-3-3s in both phosphorylation-dependent and -independent manners. The existence of both phosphorylation-dependent and -independent interactions could serve to enhance the interaction, maintain the interaction, or regulate the different functions of their binding partners. Therefore, it is likely that 14-3-3 may interact with Rac1 in both phosphorylation-dependent and phosphorylation-independent manners; however, both interactions require the presence of S71. Mutating S71 to A completely abolishes its interaction with 14-3-3s and phosphorylation of S71 strongly enhances its interaction with 14-3-3s.

It is well established that 14-3-3s are scaffold proteins and function to regulate the activity of their binding partners [2]. On the other hand, Rac1 is a GTPase and functions as a switch to directly control many signaling pathways. Thus, it is likely that 14-3-3s serve to regulate the activity and function of Rac1. Indeed, we showed that disruption of the interaction between 14-3-3 and Rac1 alters Rac1 activity and Rac1 subcellular localization (Figure 3 and Figure 6). However, the activity of Rac1 has no effect on the interaction between 14-3-3 and Rac1 and the disruption of the interaction between 14-3-3 and Rac1 does not change the subcellular localization of 14-3-3.

**Figure 7:**
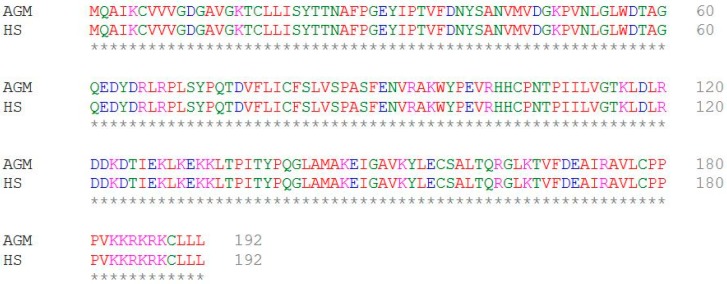
The amino acid sequence alignment of human Rac1 and African green monkey Rac1.

There are seven 14-3-3 isoforms in mammalian cells. These isoforms may have different subcellular localizations and functions [2,31,32]. We determined which isoforms interact with Rac1 and showed that three isoforms including 14-3-3η, -σ, and -θ bind to Rac1 in Cos-7 cells and 293T cells. Surprisingly, 14-3-3γ binds to Rac1 in 293T cells but not in Cos-7 cells. We do not fully understand what causes this difference. Cos-7 cells are isolated from the kidney epithelial cells of African green monkeys, and HEK-293T cells are isolated from human embryonic kidney cells. Human Rac1 and African green monkey Rac1 have an identical amino acid sequence (Figure 7). Thus, it is possible that this difference is due to other factors such as expression levels and post-translational modifications.

We have shown recently that 14-3-3η, -σ, and -θ are all associated with the cytoskeleton, while 14-3-3η also showed a strong association with the mitochondria and 14-3-3σ showed a strong association with the centrosome during mitosis [43]. 14-3-3γ mostly localized to the nucleus [43]. Rac1 has been shown to strongly associate with the cytoskeleton and regulate cytoskeleton remodeling and cell migration, in addition to the nucleus and the mitochondria [5,21,55,56]. Thus, Rac1 indeed colocalizes with these four isoforms in various subcellular locations, which support our findings that Rac1 interacts with 14-3-3η, -γ, -σ, and -θ.

The colocalization and interaction of Rac1 with specific 14-3-3 isoforms provide interesting clues and a strong basis for studying the role of this interaction in regulating specific and critical cell functions. For example, both 14-3-3 protein and Rac1 have been shown to regulate apoptosis. The interaction between 14-3-3η and Rac1 and the localization of both proteins in the mitochondria suggest that they may co-regulate mitochondria-mediated apoptosis. The colocalization of 14-3-3γ and Rac1 in the nucleus and our observed interaction between 14-3-3γ and Rac1 in 293T cells suggests that they may co-regulate certain nuclear functions.

## 5. Conclusions

**Figure 8:**
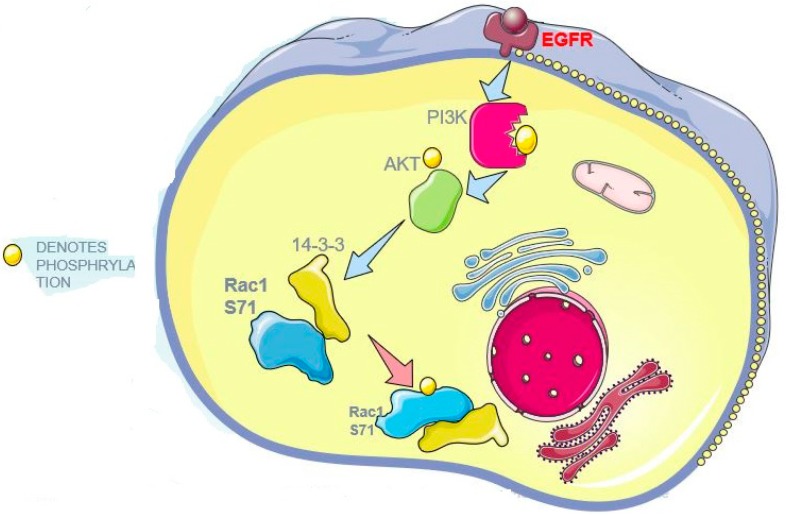
Diagram to depict the interaction between Rac1 and 14-3-3s.

We demonstrated here that 14-3-3s interact with Rac1 (Figure 8). This interaction is mediated by Rac1 S71 in both phosphorylation-dependent and -independent manners, but the phosphorylation-dependent interaction is much stronger. EGF strongly stimulates the phosphorylation of Rac1 S71 and the interaction between 14-3-3 and Rac1. Mutating S71 to A completely abolishes both phosphorylation-dependent and -independent interactions between 14-3-3 and Rac1. The interaction between 14-3-3 and Rac1 mostly serves to regulate the activity and subcellular localization of Rac1. Among the seven 14-3-3 isoforms, 14-3-3η, -γ, -σ, and -θ interact with Rac1.
